# Geometric Effects When Measuring Small Holes With Micro Contact Probes

**DOI:** 10.6028/jres.116.006

**Published:** 2011-04-01

**Authors:** Jack Stone, Bala Muralikrishnan, Chittaranjan Sahay

**Affiliations:** National Institute of Standards and Technology, Gaithersburg, MD 20899-0001; University of Hartford, 200 Bloomfield Ave., West Hartford, CT 06117

**Keywords:** coordinate measuring machine, mechanical filtering, micro-feature, probe-tip compensation

## Abstract

A coordinate measuring machine with a suitably small probe can be used to measure micro-features such as the diameter and form of small holes (often about 100 μm in diameter). When measuring small holes, the clearance between the probe tip and the part is sometimes nearly as small as other characteristic lengths (such as probe deflection or form errors) associated with the measurement. Under these circumstances, the basic geometry of the measurement is much different than it is for the measurement of a macroscopic object. Various geometric errors are greatly magnified, and consequently sources of error that are totally irrelevant when measuring macroscopic artifacts can become important. In this article we discuss errors associated with misalignment or non-orthogonality of the probe axes, probe-tip radius compensation, and mechanical filtering.

## 1. Introduction

In recent years there has been much interest in the measurement of micro-features—with characteristic dimensions on the order of 100 μm or less—using coordinate measuring machines (CMMs) [1]. Often there is a need to measure a micro-hole using a probe that is nearly as big as the hole, with very little clearance between the probe tip and the side of the hole. When the clearance is small, the center of the stylus tip traces out a very small circle as the measurements are made, with a traced diameter much smaller than the diameter of the hole and comparable in magnitude to other lengths (such as probe deflection or form errors) which characterize the measurement process. Geometric considerations for a microhole measurement are thus significantly different than for a typical macroscopic measurement. We will discuss how several effects—probe radius compensation, mechanical filtering, and probe misalignment—impact CMM measurements under low-clearance conditions. Particularly surprising is the sensitivity of these measurements to misalignment between machine axes and the measurement axes of an analog probe [2, 3]. Misalignment errors that are entirely negligible for macroscopic measurements can exceed 0.1 μm when clearances are small; this is still a small error by most standards, but if left uncorrected it would be larger than our entire uncertainty budget for measurements of small holes at the National Institute of Standards and Technology (NIST) [2, 3]. Most CMM users will be unaware of the potential danger, which might never have noticeable effects until micro-hole measurements are attempted. Similarly, there are effects related to probe tip compensation and mechanical filtering that are primarily manifested under low-clearance conditions. It is our intent in this article to raise awareness of these effects.

In this article we consider only two-dimensional measurements, and the primary focus of the article is measurement of the diameter and form of nominally circular holes. For purposes of illustration we provide numerical examples for measurements of a 100 μm diameter hole performed with a 90 μm diameter probe. This is an extreme example, where the clearance between the probe and the wall of the hole is only 5 μm. These examples are intended not so much to be “typical” as to serve as cautionary tales. However, the examples are not necessarily unrealistic because there is always a strong temptation to measure the smallest possible hole with whatever probe is available. Throughout the article we give approximate formulas to provide quick guidance for a user who wishes to determine if the errors are likely to be important in some particular measurement situation.

This article is primarily concerned with errors of an analog probe that measures along two axes, similar to the NIST fiber probe or to probes based on flexures along two nominally perpendicular directions. Any probe which measures deflections in the xy plane will be potentially subject to misalignment errors, but the discussion of non-orthogonality errors is directly relevant only to probes designed around a Cartesian geometry. (Analogous angle-dependent sensitivity variations might occur in other types of probes but would not have the same angular dependence as modeled here). The discussion of probe compensation issues might be relevant to any kind of probe, although the basic assumption is that we are working with an analog probe.

## 2. Misalignment and Non-Orthogonality of Probe Axes

Errors associated with misalignment and non-orthogonality of the probe axes has been discussed in previous articles describing the fiber probe system used at NIST [3]. In this section we expand the previous discussion and consider interactions of various error sources. We begin, in Sec. 2.1, by discussing the simplest situation, where the probe axes are orthogonal to each other and the CMM drives the probe perpendicularly into a surface. In Sec. 2.2 and 2.3 we extend this analysis to two cases where the probe deflection is not parallel to the surface normal. Section 2.4 gives numerical results for measuring a small hole. Non-orthogonal probe axes are discussed in Sec. 2.5. Section 2.6 discusses circumstances where interactions between various error sources can give rise to errors that are significantly larger than the errors discussed in Ref. [3].

### 2.1 Basic Geometric Considerations

**Fig. 1:**
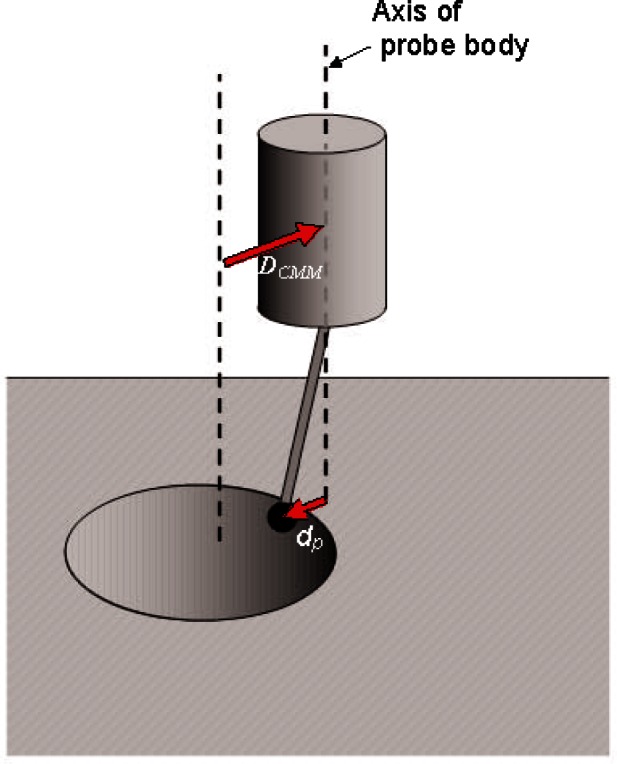
Probe deflection in contact with a surface. The diagram shows a probe contacting the side wall of a hole, just below the lip. The spherical tip of the probe, in contact with the surface, is deflected by *d_p_* relative to the axis of the probe.

When using a CMM with an analog probe, the normal method of measuring the location of a point on a surface is to move the CMM so that the probe is deflected by contact with the surface. The probe body is moved relative to the workpiece through some displacement ${\vec{D}}_{CMM}$ that first brings the probe tip into contact with the surface and then overtravels so that the probe is bent backward with vector deflection ${\vec{d}}_{p}$, as shown in Fig. 1. Adding the vector displacements ${\vec{D}}_{CMM}$ and ${\vec{d}}_{p}$ gives the position of the tip of the probe ball relative to its initial position before executing the move ${\vec{D}}_{CMM}$. The measured position of the center of the probe tip is:

(1) $${\vec{D}}_{meas}={\vec{D}}_{CMM}+{\vec{d}}_{p}.$$

**Fig. 2:**
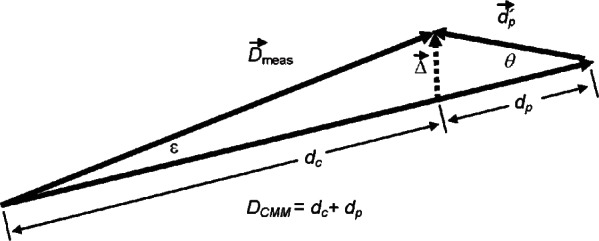
Vector diagram: machine and probe displacements.

An error will occur if the probe axes are unknowingly misaligned with the machine axes. In general, there are problems associated with both non-orthogonality of the probe axes and rotational misalignment of the probe axes with the machine axes, but to illustrate the fundamental nature of the problem as clearly as possible, we first assume that the axes are orthogonal and consider only the effect of misalignment—a rotation of both probe axes relative to the machine axes by an angle *θ*. This will occur, for example, if a perfectly constructed probe is mounted at an incorrect angle on the ram of the CMM. Then the apparent direction of the vector ${\vec{d}}_{p}$ will be rotated, and consequently the position of the center of the probe ball as given in Eq. (1) will be in error. The vector addition is shown in Fig. 2. *D_CMM_* (the magnitude of the vector ${\vec{D}}_{CMM}$) is shown as the sum of two terms, a distance *d_c_* that the probe travels before coming into contact with the surface (the clearance distance) and the magnitude of the probe deflection *d_p_*. The measured deflection of the probe is vector ${\vec{d}}_{p}^{\prime }$ with the correct magnitude $\left(d_{p}^{\prime }=d_{p}\right)$ but appearing to be tilted by *θ* relative to the true direction of probe displacement. The apparent vector location of the center of the probe is ${\vec{D}}_{meas}={\vec{D}}_{CMM}+{\vec{d}}_{p}^{\prime }$. The dashed vector $\vec{\Delta }$ is the error in the computation. When *θ* and ε are small angles,

(2) $$\Delta \approx \theta \;d_{p}$$

and

(3) $$\varepsilon \approx \theta d_{p}/d_{c},$$

where *ε* is the angular error in determining ${\vec{D}}_{meas}$.

The vector $\vec{\Delta }$ is the fundamental measurement error. Most often, the CMM will drive the probe perpendicularly into a surface, and then $\vec{\Delta }$ is nearly tangent to the surface. Consequently, the tip of ${\vec{D}}_{meas}$ will lie very close to some point on the surface to be measured, and the measurement error (the distance away from the surface) will be second order in the misalignment *θ*.

For measurements of circular geometry the most important quantity will be the error in radius measurement, *δr*. If we assume that the probe is displaced radially from the center of the circle to a point of contact with the surface, the error will be as shown in Fig. 2. The error *δr* is the error in the magnitude of ${\vec{D}}_{meas}$, given by

(4) $$\delta r={\left[{\left[D_{CMM}\;-d_{p}\;\cos(\theta )\right]}^{2}+{\left[d_{p}\;\sin(\theta )\right]}^{2}\right]}^{1/2}-d_{c}\approx \theta^{2}d_{p}^{2}/(2d_{c})+d_{p}\theta^{2}/2.$$

The approximation in (4) is valid in the limit of small *ε* and small *θ*. The first term above, *θ*^2^ $d_{p}^{2}/(2d_{c})$, is the effect of the component of $\vec{\Delta }$ that is perpendicular to ${\vec{D}}_{CMM}$ (the component tangent to the circle). The second term, *d_p_θ*^2^/2, is associated with the parallel component of $\vec{\Delta }$ and is often of less importance because it is independent of *d_c_* and hence is absorbed partially or entirely into probe calibration, as discussed later. For many purposes we can ignore this second term and approximate *δr* using its first term only, which can be thought of as a small-angle cosine error associated with angle *ε*:

(5) $$\delta r\approx (1/2)d_{c}\varepsilon^{2}\approx \theta^{2}d_{p}^{2}/(2d_{c}).$$

However, we should note that the second factor in (4) plays a role in understanding other effects to be discussed in following sections.

The error *δr* as expressed in (5) becomes negligibly small for macroscopic measurements; it is almost always of sub-nanometer order even if *d_c_* is as small as 1 mm! This minuscule error in the magnitude of ${\vec{D}}_{meas}$ and the small angular error *ε* have no practical consequences for macroscopic measurements. But when the clearance *d_c_* decreases from, say, 10 mm to 10 μm, the errors *ε* and *δr* increase by a factor of 10^3^ (and *fractional* radial errors *δr*/*r* can increase by a much larger factor). When *d_c_* becomes extremely small, the approximations used in Eq. (3), (4), and (5) are not valid, but the formula provide a reasonably good estimate of error for most situations of practical interest.

### 2.2 Measurements With a Flexible Probe

In Fig. 2 it is assumed that the *true* probe deflection ${\vec{d}}_{p}$ (as opposed to the measured probe deflection ${\vec{d}}_{p}^{\prime }$) is antiparallel to ${\vec{D}}_{CMM}$. This would be the case for a rigid microprobe, but there are complications if measurements are made using a long, thin fiber probe such as the NIST probe [2–4]. The probe is extremely flexible and consequently it is subject to electrostatic forces and vibration that can deflect it to one side. If the probe is deflected to the side when it comes into contact with the surface, it will stick at this position due to interactions with the surface and with adsorbed water films on the surface (capillary forces). Consequently, the actual point of contact with the surface will not be as intended; it will not lie along ${\vec{D}}_{CMM}$ and consequently the probe deflection will include a component that is not parallel to ${\vec{D}}_{CMM}$ or to the surface normal. Also, the angle at which the probe comes into contact with the surface will not be as intended.

It should be very clear that the sideways deflection of the probe does not, of itself, cause any errors; the probe *xy* readings should correctly account for any sideways deflection of the tip, so the measured coordinates of the probe tip are always correct even though the measurement point is not quite as intended. However, the sideways deflection of the probe can magnify other sources of error (such as probe misalignment).

**Fig. 3:**
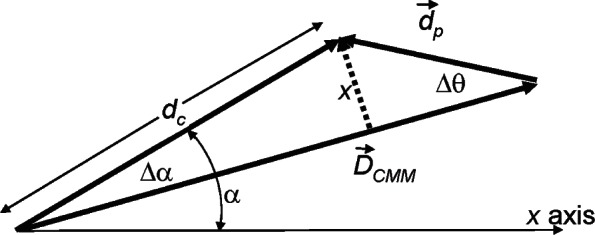
Angles associated with a measurement using a flexible probe that sticks to a surface at a distance *x* away from the intended point of contact. For purposes of this illustration there is no assumption that the probe is misaligned. Unlike Fig. 1, this diagram shows the displacement vectors relative to the CMM coordinate system. The true measurement angle *α* relative to the CMM *x*-axis differs from the direction of the approach vector by Δ*α*. The true probe deflection, ${\vec{d}}_{p}$, is at an angle Δ*θ* relative to the approach vector.

Figure 3 shows the geometry when a flexible probe is deflected to the side by an amount *x* perpendicular to the line defined by ${\vec{D}}_{CMM}$. The true deflection ${\vec{d}}_{p}$ (not ${\vec{d}}_{p}^{\prime }$ as shown in Fig. 2) is at an angle Δ*θ* = sin^−1^(*x*/*d_p_*) relative to ${\vec{D}}_{CMM}$. Also, the measurement direction *α*—the direction of the displacement of the probe tip relative to the positive *x*-axis of the CMM—is not the presumed direction, and this will have a bearing on later discussions. The actual measurement direction *α* differs from the expected measurement angle by Δ*α* = sin^−1^(*x*/*d_c_*).

To analyze the combined effect of a flexible probe and probe misalignment, a rotated probe vector ${\vec{d}}_{p}^{\prime }$ could be drawn in Fig. 3 and $\vec{\Delta }$ analyzed as before. For small angles *θ* and Δ*θ*, an equivalent method of analysis is to replace *θ* with (*θ* + Δ*θ*) in Fig. 2 and in Eq. (4). With this substitution in Eq. (4), *δr* is proportional to (*θ* + Δ*θ*)^2^, giving (a) terms proportional to Δ*θ*^2^ which should not be interpreted as an error because they belong in the calculation of the radial distance when the probe is deflected to the side, (b) terms proportional to *θ*^2^ which are the alignment error discussed previously, and (c) new terms proportional to 2*θ* Δ*θ* depending on both Δ*θ* and on the probe misalignment *θ*. The new terms 2*θ* Δ*θ* are often larger than the *θ* ^2^ terms discussed previously.

For small angles, this analysis gives the original error of Eq. (4) plus an additional error of magnitude

(6) $$\delta r^{\prime }\approx \theta \Delta \theta (d_{p}+d_{p}^{2}/d_{c})=d_{p}\theta \gamma$$

where *γ* = Δ*α* + Δ*θ* is the angular misalignment between the true probe deflection and the radial direction to the point of contact (along ${\vec{d}}_{c}$). When Δ*θ* exceeds *θ*/2, *δr*′ will be larger than *δr* and should not be ignored.

Whereas in Fig. 2 $\vec{\Delta }$ is nearly perpendicular to the radial direction (the surface normal), with the flexible probe it may be rotated through the angle *γ* and thus develop a component along the radial direction; Eq. (6) can be simply interpreted as arising from this radial component. Another situation where $\vec{\Delta }$ (and ${\vec{d}}_{p}$) is rotated is described in the next section.

### 2.3 Off-Center Measurements of a Small Hole With a Misaligned Probe

**Fig. 4:**
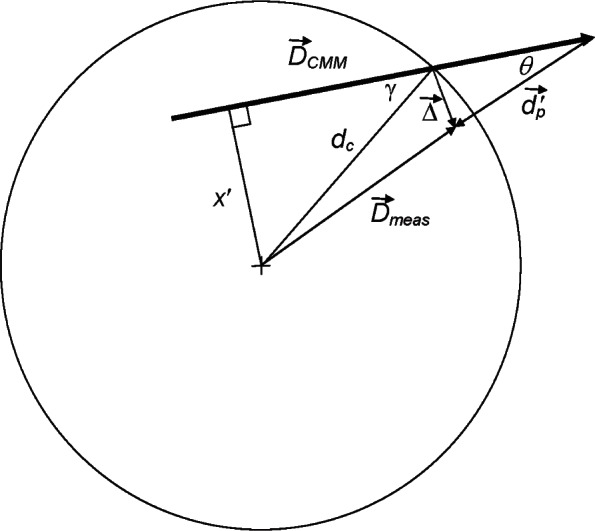
Schematic representation of the off-center measurement of a hole. The CMM displacement ${\vec{D}}_{CMM}$ (heavy vector) starts at a position that is not at the center of the hole. For clarity, the probe misalignment angle *θ* has been drawn in the opposite direction from what was shown in Fig. 2.

Consider explicitly the case of using a probe of radius *r_p_* to measuring a small hole with radius *r_h_*. The true errors of interest are the vectors $\vec{\Delta }$ at each measurement point, where the measurement points are distributed on a circle with a radius *d_c_* = *r_h_* – *r_p_*. We can analyze the radius errors using Eq. (4), assuming that the probe executed a fictitious motion from the center of the circle. However, if the actual measurements are made relative to a point that is not quite at the center of the circle, then the probe will not drive radially into the surface, the angle between $\vec{\Delta }$ and the radius vector will change, and new errors arise in a manner entirely analogous to what was shown in Fig. 3. Figure 4 shows the geometry when the CMM displacement does not start in the center of the circle and there is an error $\vec{\Delta }$ of magnitude Δ ≈ *θ d_p_* due to misalignment of the probe. When we analyze the effect of $\vec{\Delta }$ on measuring the hole radius, in addition to the error of Eq. (4) there is an additional error with a magnitude that is again given by Eq. (6), with magnitude |*δr*′| ≈ *d_p_θγ* (for small *θ* and *γ*), where *γ* is the misalignment between the true probe deflection and the radial direction to the point of contact (along ${\vec{d}}_{c}$) as in Sec. 2.2. The result is the same as for the flexible probe even though the physical interpretation is different. For this off-center measurement (unlike the flexible probe), *γ* is also equal to the angular misalignment between ${\vec{D}}_{CMM}$ and the radial direction, as shown in Fig. 4. Equivalently, it depends on the offset *x*′ shown in the diagram: *γ* = sin^−1^(*x*′/*d_c_*). For off-center measurements the error in measurement direction, Δ*α*, is simply Δ*α* = *γ*.

Thus the effect of the off-center measurement is much the same as that of the flexible probe discussed previously. However, when we measure the entire circle from a single off-center point, the radial errors *d_p_θγ* vary in a predictable manner as a function of measurement angle, unlike the case of the flexible probe where errors vary unpredictably from one point to the next. For an off-center measurement of the complete circle, the errors *δr*′ are negative in regions where the vector $\vec{\Delta }$ is rotated toward the center of the circle (such as depicted in Fig. 4) and positive in other regions; the net result is that there is essentially no change in the average radius, but the center of the best-fit circle is shifted by approximately *d_p_θγ*_max_ where *γ*_max_ is the maximum value of *γ*. (It is assumed that the magnitude of probe deflection, *d_p_*, is the same for all measurement points.) Off-center measurements also cause spurious form error measurements. The apparent form errors are a strong function of the off-center distance. They are smaller than the shift in the center position unless the off-center distance is more than ≈70 % of the clearance.

We will discuss these errors further in Sec. 2.6. For the moment we will assume that the CMM moves along a radial direction without off-center or flexible probe effects and consider the effect of misalignment only.

### 2.4 Measuring a Small Hole With a Misaligned Probe: Basic Considerations

The probe tip literally measures a circle of radius *d_c_*, and from this we must infer the actual radius and form of the hole. For a perfectly circular hole, we simply add the probe radius to the measured value of *d_c_* in order to determine the radius of the hole. (See Sec. 3 for a discussion of imperfect geometry.) Therefore it is first necessary to calibrate the effective probe diameter by using the probe to measure the known diameter of a calibration sphere. The measured diameter of a hole then depends on the difference between errors in the calibration process and errors when measuring the hole. As mentioned previously, errors independent of *d_c_* cancel out. The second term on the right in Eq. (4), *d_p_θ*^2^/2, will be of little importance if the probe deflection *d_p_* is nearly the same during calibration and when measuring a part. After probe calibration, the effect of this term is merely to change the effective diameter of the probe tip. In fact, depending on the details of how the probe is calibrated, the term *d_p_θ*^2^/2 may vanish entirely. Previously we assumed that the probe correctly read the *magnitude* of the deflection, although the angle was in error. This is not always the case. We usually calibrate the probe sensitivity in situ, mounted on the CMM; the sensitivity of the *x* and *y* probe axes are calibrated by driving the probe along *x* or *y* through known displacements into a surface and thus determining a probe sensitivity calibration along the two axes. If the probe is misaligned at the time of calibration but this is not explicitly taken into account, then the probe calibration will be in error by a cosine factor, *d_p_θ*^2^/2, and this error in calibration will just cancel the *d_p_θ*^2^/2 term in Eq. (4). ($\vec{\Delta }$ will be exactly perpendicular to ${\vec{D}}_{CMM}$ in Fig. 2).

Note that errors that depend on *d_c_* will be important even if the calibration sphere has the same diameter as the hole to be measured; for the external diameter measurement, *d_c_* should be interpreted as the radius of the circular locus of measurement points—the distance from a measurement point to the center of the sphere (*d_c_* = *r_s_* + *r_p_* where *r_s_* is the sphere radius). This can be much greater than *d_c_* for the hole (*d_c_* = *r_h_* − *r_p_*) even though *r_s_* = *r_h_*.

**Fig. 5:**
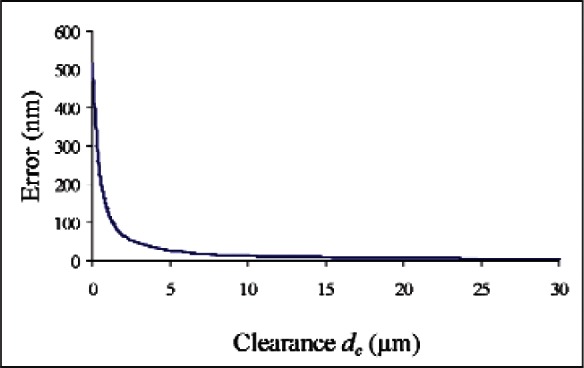
Error in radius measurement due to probe misalignment.

Figure 5 shows the overall error in measuring the distance to the wall of a hole (assumed to be perfectly round) as a function of the clearance *d_c_*. These results were calculated for *d_p_* = 15 μm, *θ* = 2°, a probe diameter of 90 μm, and assuming that the probe was calibrated by measuring the equatorial diameter of a 2-mm diameter ball, so that *d_c_* = (90 μm + 2 mm)/2 = 1045 μm for the calibration measurement.

For clearance (*d_c_*) values of most interest—the range from a few micrometers to about 100 μm—the error as shown in Fig. 5 is well approximated by Eq. (5). The angular error *ε* has no effect on this measurement.

For a 100 μm diameter hole measured with the 90 μm diameter probe (*d_c_* = 5 μm), the error in a radius measurement is 27 nm, giving an error of 54 nm for the diameter measurement. In the limit of zero clearance (*d_c_* → 0), the diameter error is approximately 1 μm.

For typical macroscopic dimensions the diameter errors shown in Fig. 5 are truly negligible by any standard, ranging from 0.4 nm at *d_c_* = 0.4 mm to −0.26 nm for infinite *d_c_*. Without probe calibration, the error at infinite *d_c_* would be 9 nm, arising from the second term in Eq. (4).

The apparent position of every measurement point on the circle is rotated by the constant angle *ε*, which becomes quite large as *d_c_* decreases. This has no effect for perfectly round artifacts, but if the hole has an imperfection, the angular location of the measured imperfection will be shifted from its true orientation. Also, there are potential problems if the probe tip itself has poor form. Normally we calibrate the probe form against a good calibration sphere and then apply corrections as a function of the measurement angle. The calibration against a macroscopic sphere will have no appreciable angular errors, but angular errors when measuring the small hole will cause the corrections for probe form to be applied at the wrong angle. In most circumstances the effect is not of major importance, but it could be significant if the probe ball has exceptionally poor form (as sometimes occurs for microprobes). For example, if the 90 μm diameter probe is elliptical by 0.5 μm and all other parameters are as stated previously (*d_c_* = 5 μm, *d_p_* = 15 μm, *θ* = 2°) then errors in radius measurement will vary between +26 nm and –26 nm as a function of angle; that is, a perfectly round hole will appear to have 52 nm of form error.

It might be possible to avoid this error if corrections are made based on the angle of the CMM approach vector rather than on the basis of the calculated angle. However, this method is probably not viable under low-clearance conditions. When using a flexible probe or with the center of the hole imperfectly known, the angular errors Δ*α* are often greater than the *ε* errors in the calculation of *α*.

Finally, we note that micro-holes such as fuel injectors can be expected to be out-of-round by at least 1 μm, and this complicates matters because now *d_c_* is a function of angle. However, the effects of this complication are small relative to the total out-of-roundness and thus not a major concern.

### 2.5 Non-Orthogonal Axes

Non-orthogonal probe axes are a generalization of the misalignment discussed previously. Consider a situation where the *x*-axis is misaligned by *θ_x_* and the *y*-axis by *θ_y_*, where the two angles are equal for the case described previously but *θ_x_* ≠ *θ_y_* when the probe axes are non-orthogonal. For non-orthogonal axes, the measurement error will be a function of the angle of measurement, giving rise to an apparent form error in addition to errors in the average diameter.

If the actual components of the vector ${\vec{d}}_{p}$ in the machine coordinate system are *p_x_* and *p_y_*, the measured components $p_{x}^{\prime }$ and $p_{y}^{\prime }$ will be

(7) $$\left(\begin{array}{c} p_{x}^{\prime }\\ p_{y}^{\prime } \end{array}\right)=\left(\begin{array}{cc} \cos(\theta_{x}) & \sin(\theta_{x})\\ -\sin(\theta_{y}) & \cos(\theta_{y}) \end{array}\right)\left(\begin{array}{c} p_{x}\\ p_{y} \end{array}\right).$$

**Fig. 6:**
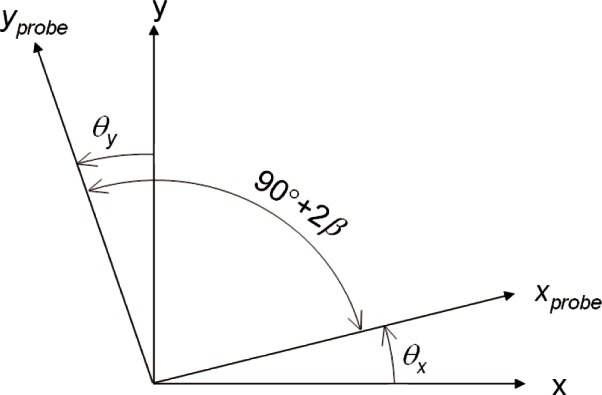
Definitions of *θ_x_*, *θ_y_*, and the non-orthogonality 2*β*. The *x* and *y* axes are the CMM machine axes.

The sign convention in the formula above is that angles are positive when the probe axis is rotated counter-clockwise away from the machine axes, as shown in Fig. 6. For *θ_x_* ≠ *θ_y_* the probe axes are non-orthogonal. Errors due to probe misalignment and non-orthogonality can be calculated in a straightforward manner if ${\vec{D}}_{CMM}$ and ${\vec{d}}_{p}$ are split into components and Eq. (7) is used to determine ${\vec{d}}_{p}^{\prime }$.

It is also convenient to introduce new angular variables that separate non-orthogonality from rotational misalignment. Let *θ* be the counterclockwise rotation of the probe axes relative to machine axes, and let 2*β* be the non-orthogonality between axes:

(8) $$\beta =(\theta_{y}-\theta_{x})/2,$$

(9) $$\theta =(\theta_{y}-\theta_{x})/2.$$

The angle *β* is positive if the angle between the positive *x*-axis and positive *y*-axis of the probe exceeds 90°. For |*β* | > 0, the probe sensitivity (scale factor) is a function of the measurement angle *α*, and this has consequences for both macroscopic and microscopic measurements. The exact nature of the errors depends on how the probe sensitivity is calibrated and how data is taken. Suppose that the probe sensitivity (change in reading for a unit displacement) has been determined correctly for each probe axis individually. Then the overall sensitivity of the probe will be a function of the measurement angle; for a pure non-orthogonality with no additional alignment error (*θ* = 0), the sensitivity variation causes radial measurement errors ranging from +*β d_p_* at *α* = 135° or 315° to −*β d_p_* at 45° or 225°. For *β* = 1° and a probe deflection *d_p_* = 15 μm, the errors at these angles are quite large—about ± 260 nm. However, the effect can be mitigated if probe calibration is done as a function of *α*, so that the error that occurs when measuring the calibration ball subtracts from the error when measuring the artifact at the same approach angle. This correction procedure is imperfect if *α* is not measured correctly (as discussed below), but if we ignore this complication, then the only errors of importance are associated with a spurious perpendicular component of the probe deflection analogous to the vector $\vec{\Delta }$ discussed previously. Unlike the case of rotational misalignment, for non-orthogonal axes the radial error is a function of *α*.

**Fig. 7:**
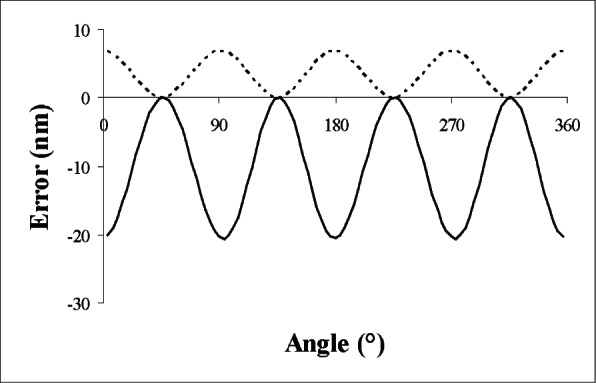
Errors with 2° non-orthogonality. (Probe *x*-axis misaligned by 1° and *y*-axis misaligned by –1°.) The dashed line shows the radial error, after probe calibration against a 2 mm diameter sphere, excluding considerations of angle measurement error. The solid line shows the error when angle measurement errors cause the wrong calibration value to be applied.

After calibration, the apparent form error of a perfectly round hole is shown by the dashed line in Fig. 7, calculate for 2° of non-orthogonality (*β* = ±1°) and other parameters as used in the previous examples (5 μm clearance). The total form error (peak-to-valley variation) is 7 nm. As in the case discussed previously, the error becomes negligibly small for measurement of macroscopic artifacts.

There is a potential for larger errors. Presumably we would apply the calibration corrections as a function of the measured *α*, as was done previously for a probe with form error. That is, if we were not aware that the probe axes were non-orthogonal, we would incorrectly interpret the apparent out of roundness of the calibration sphere as a form error in the probe tip, which should be corrected as a function of *α*. In the case of form error, the correction was not perfect because of the angular error *ε*, and an analogous error occurs with non-orthogonal axes, which give rise to errors in *α*. The resulting errors in radius measurements are shown as the solid line in Fig. 7. For a non-orthogonality of *β* and with *θ* = 0, the radial error *δr* is approximately given by

(10) $$\delta r\approx (d_{\mathrm{probe}}^{2}/d_{c})(\frac{1}{2}-A)\beta^{2}\cos^{2}(2\alpha )$$

where the factor *A* is zero for the dashed line and 2 for the solid line (the effect of including errors in *α*). This approximation is valid for typical situations and is a useful guide for understanding how the errors scale with changes in various parameters, but when careful quantitative results are needed, it is more reliable to calculate the error exactly using the method mentioned at the start of this section.

Note that the error in *average* radius is positive for the first calculation (the dashed line in Fig. 7) but negative when angular effects are included, and that the apparent form error is a factor of 3 larger when the angular effects are included. These observations are not generally true for arbitrary combinations of *β* and *θ*.

### 2.6 Misalignment and Non-Orthogonality Errors With a Flexible Probe or Incorrect Approach Vector

The error estimates developed above can significantly underestimate the full magnitude of potential problems if a flexible probe is employed, or if the approach vector is not radial because of imprecise knowledge of the center of the hole. As discussed previously, error terms of magnitude *d_p_θγ* will occur if the probe axes are rotated by *θ* and the true probe deflection differs from the radial direction by an angle *γ*, and these errors might easily exceed the basic *θ* ^2^ misalignment error. The angle Δ*θ* can be large; it will be 4° if the probe were deflected by *x* = 1 μm to the side as it contacts the surface and then drives 15 μm into the surface. Under these circumstances the *d_p_θγ* term will likely be the dominant source of error. For a clearance of 5 μm, if measurements are made from a point that differs from the true center of the hole by 0.35 μm, then the angle *γ* as shown in Fig. 4 will also be 4°, and there may again be significant errors associated with the combined effect of the off-center measurement with probe misalignment.

For the general case of non-orthogonal axes, the errors depend on both *γ* and Δ*α* in combination with *β* and *θ*. The most important effect is that the measurement angle *α* does not correspond to the direction of probe deflection; the non-orthogonality error is actually a function of probe deflection angle, but we are misinterpreting it as a form error, which is a function of *α*, and the correction process is imperfect when *α* does not correspond to the probe deflection angle. For a difference of 4° between these two angles, the maximum error as shown in Fig. 7 could rise from 20 nm to nearly 60 nm. Furthermore, this error is not uniquely associated with low-clearance conditions. Although Δ*α* is usually small when *d_c_* is large, for flexible probes the angle Δ*θ* can be large regardless of the clearance, and consequently the measurement direction is not the same as the probe deflection direction. These are large errors that can be avoided as described in the next section.

### 2.7 Mitigating Errors From Misaligned Probe Axes

As seen in the preceding discussions, probing errors due to misaligned/non-orthogonal axes can represent a significant danger for micro-hole measurement if the user is unaware of the problem and has not taken steps to correct it. However, errors associated with misalignment are unlikely to be significant if it is possible to align the probe axes so as to reduce *θ_x_* and *θ_y_* to less than 0.2°, even if the effects of a flexible probe and incorrect approach vector are considered. Alternately, these errors can be compensated via software correction if the angles *θ_x_* and *θ_y_* are known from ancillary measurements [2]. For the probing system used at NIST the alignment / orthogonality errors can be quite large—as large as 5°—but software corrections based on Eq. (7) are effective in eliminating much of the error.

The complications of a flexible probe or incorrect approach vector need not be considered explicitly in the software correction. Eq. (7) properly accounts for probe errors regardless of whether the probe deflection is in the expected direction or not.

After software correction, residual uncertainty occurs only as a consequence of uncertainties in measuring the misalignment angles *θ_x_* and *θ_y_*. The angles may be measured by displacing the CMM along its *x* or *y* axis while the probe is in contact with a surface nominally perpendicular to the direction of motion [2]. If desired, non-orthogonality can be measured directly by comparing the apparent sensitivity of the probe (scale factor) at 45° and at 135°.

Even after software correction, greatly misaligned axes do entail some magnification of uncertainties; errors scale as *β*^2^ or *θ*^2^ and consequently the uncertainty after software correction scales as *β* d*β* or *θ* d*θ* where d*β* and d*θ* are the uncertainties in the two angle measurements. Consequently, uncertainties are lower if the alignment errors are adjusted to make *β* and *θ* as small as possible. However, there will also be error terms proportional to various combinations such as *γ* d*θ* or Δ*α* d*β*, so there is not great advantage in adjusting *θ* or *β* to be much smaller than typical values of *γ*. Even with various angular errors (2*β*, *θ*, Δ*θ*, Δ*α*, and *γ*) as large as 5°, it is not difficult to keep the final uncertainty in the nanometer range. For example, if *d_p_* = 15 μm, *d_c_* = 5 μm, and 2*β* = *θ* = 5°, the errors range from +220 nm to –127 nm before correction. But if the angles *θ* and 2*β* are known with an uncertainty of 0.05° (which is practical to achieve), then the uncertainty of the measurement results after correction is less than 10 nm at any angle, including effects associated with Δ*θ*, Δ*α*, and *γ*.

Equation (7) does not include the effects of probe form error, which must be corrected based on measurements of the calibration sphere. When measuring form error against the calibration sphere, it is necessary to first correct for non-orthogonality using Eq. (7). It might seem logical to absorb the non-orthogonality error into an “effective form error” because it is independent of *d_c_*, but this is unadvisable. The non-orthogonality error is actually a function of probe deflection angle, whereas the form error is a function of *α*, and these two angles are not always equal as seen in Fig. 3.

An alternate approach to correcting misalignment/non-orthogonality errors is to take readings at several different probe deflections and extrapolate to zero deflection. Note that a linear extrapolation is not appropriate because misalignment errors scale proportional to $d_{p}^{2}$, as do non-orthogonality errors if *α* were recomputed at every deflection. Additional complications occur when using a flexible probe, where the probe deflection may not lie along the same direction as the CMM approach vector. One procedure that might be employed under these circumstances is to drive into contact with the surface, read the probe, and, based on the probe reading, back off the CMM to the point where the expected probe deflection is zero (not necessarily along the approach vector). At this point the probe has some small deflection *d_p_* which is measured and used to compute the final result. If *d_p_* is reduced from 15 μm to < 1 μm, errors proportional to $d_{p}^{2}$ would become negligibly small. In general, errors will be substantially reduced, although some of the expected improvement may be counteracted by imperfect machine control that will likely causes Δ*θ* (in Fig. 3) to increase as ${\vec{d}}_{p}\to 0$. It is of interest to note that, under normal circumstances, there is no danger in attempting to drive ${\vec{d}}_{p}$ to zero even if there is a possibility of slightly overshooting the surface so that ${\vec{d}}_{p}$ switches direction; in most cases a microprobe will stick to the surface after making contact and will still give a valid reading when the probe is retracted by a small amount.

## 3. Surface Reconstruction: Probe Tip Compensation and Mechanical Filtering

From the measured position of the center of the probe at each measurement point it is necessary to infer the actual location of the surface. Problems arise in relating the position of the center of the probe to the actual point of contact (probe tip compensation) and additional problems arise when the probe is too large to touch portions of the surface (mechanical filtering).

### 3.1 Methods for Probe Tip Compensation

Probe tip compensation (stylus radius correction) is not usually a problem when measuring simple shapes of macroscopic size, but it can be a problem for microprobes when clearances are small. In this section we cannot discuss all aspects of probe compensation but wish to bring attention to some effects that are uniquely important for measurements with microprobes.

From the measured position of the center of the probe, it is necessary to infer the position of a point on the surface being measured. Exact compensation for the probe tip radius requires that the measured coordinates of the center of the probe be increased by a vector whose magnitude is equal to the probe radius and whose direction goes from the measured coordinates of the center of the probe to the point of contact with the surface. There is often an ambiguity as to the location of the point of contact. For a typical CMM measurement, this presents no problem when measuring a nominally circular hole. The hole can first be measured ignoring compensation, its center point determined via best fit, and then compensation applied at each measurement angle by adding the probe radius to the measured radius at that angle. That is, the direction of contact is assumed to lie along the radial direction of the best-fit circle. Below we will refer to this simplest technique as “radial compensation.” Radial compensation always underestimates the true magnitude of the hole radius at any given angle. In fact, for any method of compensation where a probe radius vector is added to the coordinates of the probe center, the apparent point of contact cannot lie within the physical material and consequently compensation errors will always result in underestimating an internal diameter or overestimating an external diameter. In the following discussion of hole measurements the compensation errors are always negative.

There is some temptation to apply probe tip compensation along the direction of the CMM approach vector, but this is not generally advisable, particularly when using a flexible probe (for which there is no guarantee that the path followed by the CMM corresponds to the path of the probe tip). Even with a rigid probe measuring a perfect circle, adding the probe radius along the approach direction may not yield accurate results. Errors will occur if the center of the circle is not known precisely so that the approach vector cannot be made to lie exactly along a radial direction, perpendicular to the surface. If ${\vec{D}}_{CMM}$ differs from the radial direction by a small angle *γ*, then the error vector $\vec{\Delta }$ will be roughly perpendicular to ${\vec{D}}_{CMM}$ (and to the radius vector) with magnitude *r_p_γ*. For our usual example of a 90 μm probe measuring a 100 μm diameter hole, if the center of the hole is incorrectly located with an error *x*′ = 1 μm, the *r_p_γ* varies as a function of measurement angle and has a maximum value of 9 μm. The resulting errors in the diameter measurement are not this large since $\vec{\Delta }$ points nearly tangentially to the circle being measured. For small clearance (*d_c_* ≪ *r_h_*) and for *x*′ ≪ *d_c_*, the error in radial compensation is approximately –(1/2)*d_c_γ*^2^ and *γ* varies between 0 and *x*′/*d_c_*. Thus the maximum compensation radial error is –(*x*′)^2^/(2 *d_c_*) and is inversely proportional to the clearance. For the parameters given above, the maximum radial error is ≈–100 nm and the minimum error is 0, giving rise to ≈100 nm of apparent form error. The average apparent diameter would also be in error (too small) by roughly 100 nm. This potential error is avoided when compensation is applied along the radial direction of the best-fit circle rather than along the approach vector.

For macroscopic measurements of a near-perfect circle, the simple technique of radial compensation is entirely satisfactory. For example, consider what happens if a hole of 10 mm diameter is measured by a 2 mm diameter probe. A low-quality hole that has not been reamed might have a form error of elliptical shape with the major axis 100 μm longer than the minor axis. Then measurement errors due to radial probe compensation range from 0 to –62 nm as a function of the measurement angle. Although a 62 nm radial error is not negligible for a high-accuracy measurement, it is so small relative to the total out-of-roundness (10^5^ nm) that it is unlikely to ever have practical consequences.

The problem is often more pronounced when measuring micro-holes with small clearance, and with an out-of-roundness somewhat comparable to the clearance. Even a good quality micro-hole may be out of round by at least 2 μm. For a hole with 100 μm minor diameter and 102 μm major diameter, and a probe 90 μm in diameter, the clearance ranges from 5 μm to 6 μm, which is a 20 % variation. Consequently, the eccentricity of the path followed by the center of the probe is much more pronounced than that of the actual hole, and this will magnify the errors of radial probe compensation. The errors are somewhat mitigated by the small radius of a microprobe, but errors are nevertheless significant, reaching a maximum magnitude of 80 nm at angles in the vicinity of 45° relative to the axes of the ellipse. There is no error along the major and minor axes and consequently the total out of roundness does not change, but the detailed shape of the hole is not measured correctly, and the average radius is reduced. The 80 nm errors are, in absolute terms, not so different from our estimated typical errors for a macroscopic hole, but as a fraction of the diameter the errors are 100 times greater, or as a fraction of the out-of-roundness the errors have increased by a factor of 50. Nevertheless, the errors are still only 4 % of the total out of roundness and thus are only of marginal significance.

### 3.2 Compensation Problems When Using an Imperfect Probe Tip

Radial compensation is perhaps more problematic when the *hole* has good geometry but the *probe* departs significantly from spherical. One source of compensation error, associated with probe axis misalignment in conjunction with poor probe form, was discussed in Sec. 2.4. Additional effects occur even with a well-aligned probe.

Unfortunately, the geometry of microprobes is often not of the same high quality typical of macroscopic probes with ruby tips. For example, the fiber probes used in our research are elliptical by as much as 0.5 μm around the equator and can be ten times worse for a circle passing through the pole (the direction along which the stem is attached). As a first approximation, the departures of the probe from spherical form (or departures from circular form for two-dimensional measurements) can be taken into account by mapping the probe out-of-roundness via measurements of a known, high-quality calibration sphere, thus determining corrections that can be applied at each measurement angle, using radial compensation to determine the point of contact with the surface. This does not completely solve the problem, however, because the point of contact will depart from the radial direction if the probe out-of-roundness is significant. As usual, the problem is most pronounced under low-clearance conditions. For a probe with a minor diameter of 90 μm and an major diameter of 92 μm, measuring a circular hole of 100 μm diameter, errors due to assuming radial compensation reach a maximum magnitude of about 120 nm. The error is roughly proportional to the square of the out-of-roundness and inversely proportional to the clearance.

**Fig. 8:**
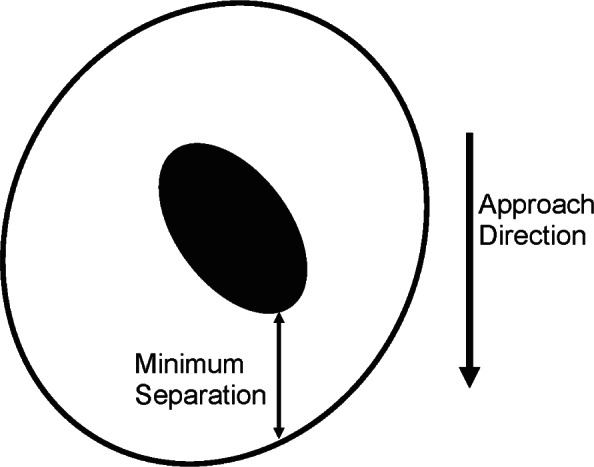
Measuring an elliptical hole with an elliptical probe. By determining the minimum separation along the approach direction, we find the point of contact between the probe and the wall of the hole.

For some measurements the probe may be more out-of-round than the part, and under these circumstances—as illustrated in the example above—simple radial compensation may result in unacceptably large errors, particularly when clearances are small. We have investigated one possible scheme for probe compensation that works well under the circumstances typical of our small-hole measurements. The basic idea is that, if the geometry of the probe and the part were known exactly, then the point of contact could be predicted, for a given approach vector, by finding the points on the two surfaces with the minimum separation between the part and the probe along the approach direction, as illustrated in Fig. 8. The minimum separation can be determined numerically regardless of the shape of the hole and the probe. We should note that, if the hole being measured is everywhere convex, then it does not matter that the true approach vector may differ from the assumed approach vector (as long as the assumed initial position of the probe at the start of its motion lies entirely within the hole).

We know the measured center coordinates of the probe when it is in contact with the surface at some measurement point. If we knew exactly the shape of the probe and the shape and position of the hole, the procedure above would correctly calculate the corresponding point of contact between the probe and the surface. In practice, the form of the probe is determined by calibrating it against a good calibration sphere, but the exact shape and position of the hole are unknown. Therefore an iterative procedure is required. We start by assuming that the hole is circular, centered at the origin, and calculate the points of contact and corresponding probe radius compensation vectors accordingly. These compensation vectors are then added to the measured coordinates of the probe center to get measurement results in first approximation, giving a better picture of the true shape and position of the hole. The calculation can then be iterated, where each iteration successively improves knowledge of the hole geometry, providing successively better estimates of the point of contact and thus of the tip compensation. The hole is first assumed to be circular but on successive iterations an ellipse can be fit if the hole is clearly elliptical. More generally, there is no reason that an arbitrary shape cannot be fit to the hole, but fitting a complex shape will make results more susceptible to noise. To filter out noise, the substitute geometry fit to the hole should contain only enough detail to capture the major geometric features.

We have simulated this process for the realistic case of an elliptical probe in an elliptical hole where both have eccentricities less than 40 %. For this situation the calculation yields accurate answers with three or four iterations. Simulations indicate that the calculation is not excessively sensitive to measurement noise.

This compensation method is appropriate for sparse data sets obtained in non-scanning measurements and is easy to implement for situations of interest in this paper (two-dimensional measurements of near-circular artifacts). For dense data sets (such as scanning measurements) more powerful techniques are available and can be employed successfully even for free-form surfaces. One technique that can be applied to any free-form, unknown surface is to acquire sufficiently dense data so that a surface can be defined by the positions of the center of the probe in contact with the part at closely spaced points. A normal to this surface can be determined at each measurement point, and the compensation vector is then applied along the direction of the surface normal. In principle, projecting the radius along the surface normal will correctly reconstruct the surface at all points that can be physically reached by the probe; gaps in the data will appear where the probe is too big to touch all of the surface (mechanical filtering). In practice, implementation of this technique for measurement of freeform surfaces in the presence of noise involves complexities well beyond the scope of this article. (See, for example, Woźniak et al. [5] for many references and a brief summary of research on this problem.) An alternative technique often used with scanning probe microscopes is “envelope reconstruction” [5-8], described mathematically as an erosion operation [7, 8]. For infinitely dense data, the envelope of the volume traced out by the probe tip reconstructs the surface at every point touched by the probe and provides a bound on the possible surface position where there are cavities too small for the probe to penetrate. Neither of these techniques is clearly advantageous for our measurements, because our data sets are fairly sparse (between 8 and 48 points around the circumference of a circle) and because we need not reconstruct completely unknown, free-form shapes.

### 3.3 Radial Compensation and Mechanical Filtering With High-Order Form Errors

The discussion of Sec. 3.1 implied that, with a perfectly round probe, radial compensation is not likely to cause important problems when measuring a nominally round hole. However, even when using a perfect probe, compensation errors become more pronounced if the same total out-of-roundness of the hole occurs at higher harmonics. These can be represented by the function

(11) $$r(\theta )=r_{h}+a[\cos(n\theta )-1]$$

where *n* = 2 will give very nearly the same results as an ellipse and higher harmonics are represented by *n* > 2. The parameter *r_h_* is the maximum radius of the wavy hole (*r_h_* = 51 μm to reproduce the previous example of the elliptical hole). Normally we expect *a* ≪ *r_h_*.

Errors are a function of angle and have maximum magnitude near the zeros of the cosine function. The maximum magnitude error as determined by exact calculations is predicted well by the empirical formula below:

(12) $$|\delta r{|}_{\max}\approx \frac{r_{p}n^{2}a^{2}}{2r_{h}d_{c}}$$

where *d_c_* is interpreted as the average clearance. (The actual error is negative, but here we are looking at the positive magnitude of the error.) The formula predicts the error reasonably well (within about 10 %) over a fairly broad range of input parameters as long as (|*δr*|_max_/*a*) < 0.6 and *a*/*r_h_* < 0.1. For input parameters that give (|*δr*|_max_/*a*) > 2, the result cannot be trusted at all because it is not even physically reasonable.

**Fig. 9:**
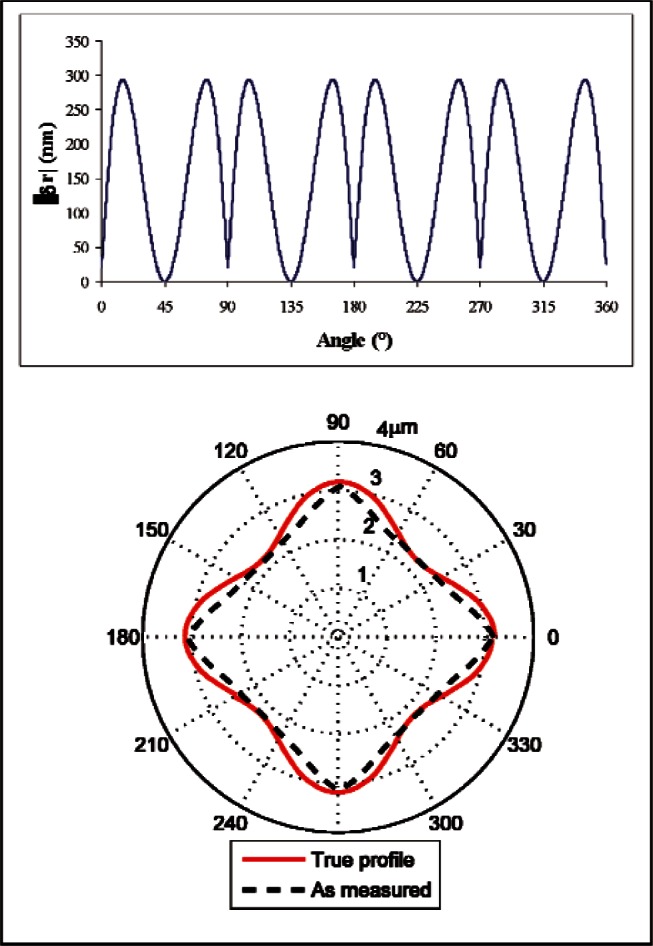
Magnitude of radial measurement errors when measuring a circle with 4th harmonic form variations. The top plot is error vs. angle. The polar plot at the bottom shows the true and measured deviations from circularity.

The errors become much larger for higher values of *n*. For *n* = 4, a simulation of the measurement process yields errors as a function of angle as shown in Fig. 9. As expected from Eq. (12), the maximum error is nearly 4 × larger than for *n* = 2 (the ellipse). The radial errors are now 290 nm, a substantial fraction of the total out-of-roundness.

A second source of error is also apparent in the simulation results. We would expect the radial compensation error to go to zero at the center of the troughs of the cosine function in Eq. (11), but this does not occur (and there are discontinuities in the slope of the curve at these points). This behavior indicates where the probe is too big to reach to the bottom of the troughs; mechanical filtering is smoothing out the indentations in the surface. The errors associated with radial compensation could be avoided by using an envelope technique, but errors due to mechanical filtering will always remain. Mechanical filtering can be a significant problem even for measurements of macroscopic holes with large clearances [9], but the problem becomes even more severe when the clearance is small, as described below.

For sufficiently high *n* in Eq. (11), the probe cannot reach the bottom of troughs of the cosine function. A necessary condition to reach the bottom of the troughs is that the radius of curvature of the probe is less than the radius of curvature of the feature being measured. The curvature, *κ*, of a curve *r*(*θ*) at a point of zero slope is given by

(13) $$\kappa =[r-(d^{2}r/d\theta^{2})]/r^{2}.$$

For Eq. (11), at the bottom of the trough located at *θ* = 0, the curvature is

(14) $$\kappa =(r_{h}+an^{2})/r_{h}^{2}.$$

To avoid mechanical filtering this curvature must be less than the curvature of the probe, which is 1/*r_p_* for a probe with radius *r_p_*. If we define the effective radius

(15) $$r_{\mathrm{eff}}=\frac{r_{h}r_{p}}{r_{h}-r_{p}}$$

then the curvature condition can be written as

(16) $$r_{\mathrm{eff}}<r_{h}^{2}/(an^{2}).$$

The right hand side of Eq. (16) is just the radius of curvature at the trough of a cosine function with amplitude *a* and a spatial wavelength 2π*r_h_*/*n*, which is the physical length of one period of the cosine function when it is wrapped around the hole diameter. The effective radius *r_eff_* is nearly equal to the probe radius when *r_h_* ≫ *r_p_*, but under low-clearance conditions (*r_h_* − *r_p_* ≪ *r_h_*) it is greatly magnified. For the case considered previously—a 90 μm probe measuring a 100 μm hole—*r_eff_* = 10*r_p_*. Hence the onset of mechanical filtering will occur at spatial wavelengths that are 10 times longer than what might first be assumed based on the comparing the probe tip radius to the radius of curvature of the cosine function. The underlying reason, of course, is that the curvature of the hole adds to the curvature of the cosine function. In this sense the result is not surprising, but it is easy to overlook this effect on first consideration. For the parameters given previously, mechanical filtering will attenuate the form error for harmonics with *n* > 2.

### 3.4 Small Protrusions: Dirt

**Fig. 10:**
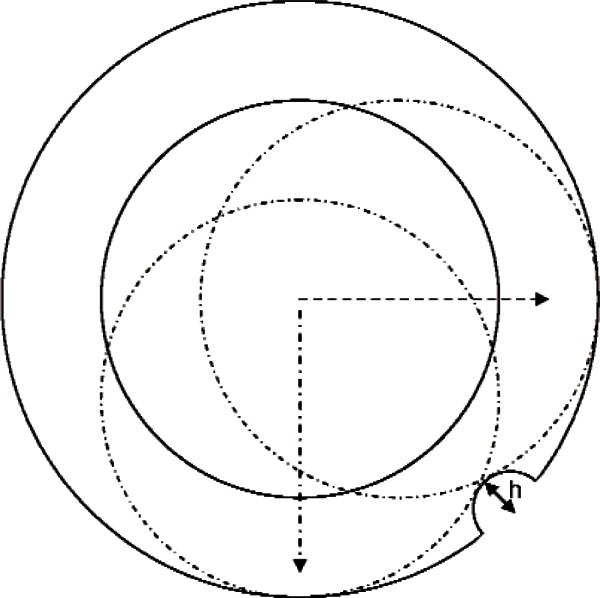
A situation where the probe will be influenced by a protrusion (of height *h*) for measurements over a broad range of angles.

Another manifestation of mechanical filtering is seen when a small protrusion is present under low-clearance conditions. Mechanical filtering will always cause a protrusion to appear wider than its true physical extent, but the probability of hitting a protrusion is higher under low clearance conditions. An example is illustrated in Fig. 10. Under low-clearance conditions, the range of measurement angles for which the probe will hit a protrusion of height *h* is approximately 2cos^−1^(1 − *h*/*d_c_*). This range is 90° for a situation such as depicted in Fig. 10, where the height of the protrusion is 29 % of the clearance. Assuming the same surface density of protrusions on a flat plane and in a hole, the probability of hitting a protrusion of height 0.29 *d_c_* when measuring the hole is enhanced by a factor of roughly $\sqrt{r_{h}/d_{c}}$ relative to measurement on a flat plane. This enhancement factor is 3 for the conditions of our usual example.

The protrusion in Fig. 10 might well be “dirt” of uncertain origin. Dirt is a particular problem when measuring long thin holes because, in addition to the effect discussed above, it is difficult to clean small holes. The problem is not particularly unique to low-clearance measurements, but it is likely to be more important for measurements of small dimensions simply because it represents a larger fractional error. Dirt is a problem for microprobes because they must be operated at very low force in order to avoid plastic deformation at the point of contact [9, 10] (because the area of contact is so small for a small probe). The NIST fiber probe operates in the micronewton regime. Traditional probes, operating at forces in excess of 0.1 N, have much less problems with dirt, even though a traditional probe with a diameter of 2 mm will have 20× greater probability of contacting a piece of dirt than a microprobe with a diameter of 100 μm. It is usually assumed that a traditional probe is not very sensitive to dirt because it can crush or push aside surface contaminants. Certainly this should be true for common forms of dirt that have structure. For example, biological structures such as a grain of pollen can be crushed even by probing forces as small as 1 mN [12]. If fibrous dust is sticking up from the surface it can often be bent toward the surface and out of the way of a traditional probe, unless the probe hits the fiber nearly directly at the point where the fiber is in contact with the surface, where there is not sufficient clearance for the fiber to be bent out of the way. The high force of a traditional probe may not be particularly helpful in reducing effects of other forms of dirt. For example, machining debris—if they took the form of compact ductile particles—will be only slightly flattened by the probe force and would not be pushed to the side. If such contaminants were indeed present, the greater force exerted by a traditional probe would not be advantageous and the larger size of a traditional probe would give a higher probability of hitting the contaminants than for a microprobe. In practice we see many more contaminant or dirt-related problems when using a microprobe than when using a traditional probe.

## 4. Other Geometries

Most of the problems discussed above are unique to small-hole measurements. As mentioned previously, errors due to probe misalignment are likely to be much more severe when measuring a small internal diameter than when measuring an external diameter of the same size. Even other measurements of internal dimensions are not necessarily subject to the same errors. For example, measuring the width of a narrow slot—if done with a reasonable measurement strategy—will not suffer the same problems as measuring a hole with comparable diameter. That is, if several measurements are taken on one face of the slot to define a plane, and if the perpendicular distance between this plane and a point on the other side of the slot is measured, the slot width will be correct in spite of misaligned probe axes (for a calibrated probe, where the second term of Eq. [4] drops out). However, if a two-point measurement is made along a pre-defined direction perpendicular to the slot, the distance between the two points will be in error by the same amount as when measuring a hole of the same diameter. For non-orthogonal axes, if the erroneous value for *α* causes the wrong scale correction to be applied, there are potential measurement errors even when using the preferred measurement strategy.

## 5. Conclusions

The point of this article is not that there are intractable problems when measuring micro-features at the nanometer level, but that one should be aware of the potential problems and take simple measures to avoid them. Many CMM users may be unaware of problems such as probe misalignment because these errors have no visible effect on macroscopic measurements and may not even affect other microscopic measurements such as slot width. Other problems, such as mechanical filtering or problems associated with radial compensation, are well known, but it may not be fully appreciated that these effects can be magnified under low clearance conditions.

All the geometric multiplication effects discussed above can be easily avoided if a suitably large clearance is maintained. When measuring a micro-hole, geometric errors can be best mitigated by employing a small probe tip, so that the clearance with the wall remains as large as possible, and so that mechanical filtering is kept to a minimum. A smaller probe tip will effectively reduce all the errors that have been discussed here—probe alignment errors, radial compensation, mechanical filtering, and the probability of contacting dirt. (On the other hand, a smaller probe may increase complications associated with surface interactions [13].) When a small tip is not available, it is important to take other measures to minimize errors as much as possible. At a minimum, it is important to measure the misalignment of probe axes and use these results for software correction, or alternately, measurements can be made at small probe deflections as described previously. Also, one should be aware of the potential errors associated with simple radial probe tip compensation, particularly if the probe itself has elliptical form; when these errors are unacceptably large, a more sophisticated compensation method should be employed.
